# A study of the improvement of academic burnout of students with different learning styles by teacher-student collaborative assessment

**DOI:** 10.3389/fpsyg.2025.1504097

**Published:** 2025-07-16

**Authors:** Yong Jiang, Wen-ting Ge, Yu-ge Wu, Han-zhu Zhou, Jun-xuan Zhang

**Affiliations:** School of Laboratory Medicine, Jilin Medical University, Jilin City, China

**Keywords:** teacher-student collaborative assessment (TSCA), academic burnout, learning style, teaching reform, student health

## Abstract

**Introduction:**

Academic burnout has become a pressing concern in higher education, particularly with the rise of online learning. This study aimed to investigate the effectiveness of the Teacher-Student Collaborative Assessment (TSCA) model in mitigating academic burnout among college students with diverse learning styles.

**Methods:**

A quasi-experimental design was applied to two undergraduate classes (*n* = 85) at Jilin Medical University. The Kolb Learning Style Inventory was used to categorize students into adaptive, assimilative, divergent, and convergent types. Academic burnout was assessed at the start and conclusion of a 3-month TSCA-based course using a validated burnout questionnaire.

**Results:**

The results indicated that students with adaptive and divergent learning styles experienced a statistically significant decrease in burnout levels (*P* < 0.05), whereas those with assimilative and convergent styles did not (*P*>0.05).

**Discussion:**

These findings suggest that the TSCA model may be particularly effective for certain learner types in reducing academic burnout and enhancing engagement in online education. This study provides practical insights for personalized teaching interventions and online course design.

## 1 Introduction

The abrupt transition to online learning during the COVID-19 pandemic heightened concerns regarding academic burnout among university students. Recent research indicates a significant increase in disengagement, mental fatigue, and reduced motivation due to prolonged screen exposure and isolation in virtual learning environments (Madariaga et al., 2023). Burnout is increasingly viewed as a multifactorial issue, shaped by both personal learning dispositions and instructional design (Yiwen et al., 2023). Self-Determination Theory (Ryan and Deci, 2000) provides a motivational lens for understanding student burnout, emphasizing the importance of autonomy, competence, and relatedness. Similarly, Kolb;s Experiential Learning Theory (1984) presents a typology of learning styles—adaptive, assimilative, convergent, and divergent—that differently influence how students engage with course material. Together, these frameworks suggest that aligning learner characteristics with pedagogical strategies may help reduce academic burnout. The Teacher-Student Collaborative Assessment (TSCA) model, which integrates teacher feedback, peer review, and self-evaluation, aligns with both constructivist and experiential principles. However, there is limited empirical evidence regarding its differential impact on diverse learning styles in a post-pandemic context. To address this gap, our study examines whether TSCA reduces academic burnout among students categorized by Kolb's learning styles. We hypothesize that TSCA significantly reduces academic burnout among adaptive and divergent learners but does not have a significant effect on convergent and assimilative learners. This research contributes to the development of personalized education strategies and theory-informed interventions that promote student wellbeing in hybrid and online settings.

## 2 Related research

### 2.1 The development of research on academic burnout

Students' learning and living environments were completely parallel; both learning and living produced unavoidable interactions, and the interactions between different states resulted in varying orientations toward academic outcomes and developments. Poor academic outcomes often began with academic burnout, which was first defined in a study by Pines et al. (1981), specifically referring to the phenomenon of energy depletion when students studied at high intensity (Pines et al., 1981). Later scholars added environmental conditions, and Meier argued that academic burnout occurred within school environments (Meier and Schmeck, 1985). Some foreign researchers have believed that students experience chronic academic stress and burden over time, depleting their energy and ultimately leading to academic burnout. This often left students in a state of emotional, attitudinal, and behavioral exhaustion due to their inability to effectively manage the negative impacts, which represented an ineffective coping strategy. However, after 2,000, some scholars posited that academic burnout was not solely due to energy depletion from excessive academic stress but also stemmed from a lack of motivation and interest in learning, supplementing the local phenomenon described by Lian et al. (2005). The specific manifestations of academic burnout included the gradual loss of enthusiasm for academics, indifference to academic performance, and alienation from teachers. Relevant studies have revealed that academic burnout often accompanies a prolonged negative stress state that induces cognitive distress (Tuominen-Soini and Salmela-Aro, 2014), leading to students' inability to effectively complete their academic tasks, a decline in academic performance, negative feedback regarding academic achievement, and procrastination, among other adverse effects.

### 2.2 Relationship between academic burnout and online courses

There was a relationship between academic burnout and online teaching, especially during the recent New Crown epidemic when many students turned to online learning because of its effects. Some research suggested that online teaching environments may increase the risk of academic burnout. Students in online classes had more autonomy, and those with less self-discipline were at greater risk of developing academic burnout due to their inability to keep up (Xu et al., 2019). The intrinsic cause of academic burnout was the lack of face-to-face interaction and classroom atmosphere that students experienced when studying at home, leading to feelings of isolation, a lack of motivation, and reduced engagement. Prolonged periods of screen-based learning also impacted students' concentration and learning efficiency. Additionally, the changes in the learning environment made students more engaged with their personal lives and more easily distracted, indicating that online teaching required greater self-management, which proved challenging for some students. The objective factors included the lack of instructional enrichment and direct supervision and guidance from instructors, which contributed to boredom and negative attitudes toward learning. However, not all students experienced academic burnout in an online environment; some found online learning to be more flexible and adaptable. Therefore, understanding individual differences and providing support and resources are vital, and gaining a deeper understanding of students' learning preferences and habits, as identified by Kolb Learning Styles, may effectively help them overcome burnout in online environments.

### 2.3 Strategies to improve academic burnout

Improving students' academic burnout is a challenge that educators must face, and various educators have attempted to address this issue through psychological interventions, teaching improvement, management strategies, and the enhancement of leisure activities. The primary improvement strategy focuses on psychological health as the starting point, teaching reform as the optimization method, and the management system along with classroom style as the means. Cultivating positive psychological capital serves as an effective strategy to mitigate students' academic burnout. Positive psychological capital acts as a mediating variable between learning motivation and academic burnout, driving learning motivation and enhancing learning interest while suppressing learning burnout (Zarezadeh et al., 2024). The constructed binding force, cohesion, and driving force based on group dynamics theory regulate academic burnout to some extent. Group cohesion is enhanced through the development of school and class styles, the optimization of management schemes, the strengthening of group relationships, and the fostering of good teacher-student and student-student relationships (Tomasev et al., 2021). Employing group dynamics theory to shape the three main forces positively promotes the construction of a healthy learning management atmosphere and environment, thereby alleviating students' academic burnout. Enriching spare time through physical exercises leads to a degree of pleasure in the brain; relaxation and enjoyment can reduce academic burnout. Research indicates that college students' academic burnout is significantly negatively correlated with physical exercise, self-efficacy, and mental toughness, and college students with high levels of physical activity demonstrate lower academic burnout than those with moderate levels, while students with low levels of exercise experience the most severe academic burnout (Chen et al., 2022).

### 2.4 Academic burnout and achievement response of different learning styles

At the end of the 20th century, David Kolb explained the significance of learning style classification through theory and suggested that teachers should pay attention to learners' learning styles. Kolb pointed out that concrete experience, reflective observation, abstract conceptualization, and active experimentation were constructed as a comprehensive learning process. Currently, most inductive explorations of learning behaviors and styles are based on the Learning Styles Inventory (Ahmad et al., 2019). Learning style is a dynamic process generated by the interaction between students and their environments; therefore, domestic researchers mainly use Kolb's Learning Style Scale to investigate and locate learners' learning styles.

Although research on different learning styles and academic burnout was limited, it was not difficult to find a correlation between learning styles and academic burnout based on previous teaching experience. Students may be more susceptible to burnout in certain learning environments where some learning elements are missing and their learning needs are not met.

Different learning styles produce varied performance responses. Compliant learners are more engaged in the classroom and demonstrate a better understanding of classroom content, thereby performing better in traditional teacher-centered teaching models (Black and Wiliam, 2009). In fact, research in this area is still in its infancy, and further studies are needed to explore the relationship between learning styles and academic burnout to provide specific guidance and support to students experiencing burnout.

### 2.5 TSCA in teaching and learning

The TSCA model has gradually emerged in instructional design and has been utilized in the teaching processes of various disciplines by integrating students' characteristics and disciplinary backgrounds to complete the assessment of learning tasks through teacher-student cooperation. Teacher-student cooperative evaluation combines teacher assessment with students' self-evaluation and peer evaluation, further merging “assessment” and “learning” under the guidance of the teacher, maximizing the use of evaluation, and viewing the evaluation process as an integral part of enhancing learning, greatly improving the efficiency of the Prastyo (2017). This teaching mode has been widely adopted in oral English teaching. Teacher-student cooperative evaluation, based on “co-assessment-mutual assessment-reflection-examination,” has played a positive role in college English listening and speaking courses and has enhanced students' evaluation skills and motivation to complete classroom tasks (Luo et al., 2023). The oral report of classical reading has been an important component of college English listening and speaking teaching, and Tang Pei proposed an optimized application plan by analyzing the evaluation process of the oral report of classical reading, demonstrating the excellent effect of the combination of teaching and learning (Luo et al., 2023). It has also been implemented in some medical or nursing education contexts, where the teacher-student cooperative teaching model has shown a strengthening effect on students' professional communication skills (Chen et al., 2023). In this study, we explored the interaction and control between student evaluation and teacher evaluation by introducing the TSCA model in the course. The students' evaluation of the teacher encouraged the instructor to make timely adjustments. Evaluation showed more positive effects during the teaching process, and Marsh et al. emphasized that student evaluation was not only valid and reliable for online teaching, but also less susceptible to bias, ultimately helping instructors identify and remedy deficiencies in a timely manner and improve their online teaching practices (Marsh, 2001). Improving two-way feedback by establishing collaborative mutual assessment between instructors and students facilitates the interactive exploration of burnout in teaching.

## 3 Objects and methods

### 3.1 Selected objects

In this study, the students of the biotechnology undergraduate class of 2019 and the undergraduate course in inspection technology of 2020 were selected as the subjects. The teaching activities of the school's online course in Clinical Molecular Biology Testing were conducted in September 2022, following the designed TSCA model of learning lattice typing and collaborative evaluation between teachers and students. For the convenience of subsequent experiments, the medical testing class of 2020 was labeled as Class A, while the biotechnology class of 2019 was labeled as Class B. The total number of valid participants was 128, including 96 students from the 2020 Clinical Laboratory Medicine class (Class A) and 32 students from the 2019 Biotechnology class (Class B). Among the participants, 70 were women (45.3%) and 58 were men (54.7%). The average age of the students was 20.6 years (SD = 0.9). All participants were undergraduate students enrolled in the Department of Medical Science at Jilin Medical University. No students reported having prior experience with TSCA or similar collaborative assessment models. This study was conducted in accordance with the ethical standards of Jilin Medical University. Ethical approval was obtained from the university's institutional review board prior to the commencement of data collection. All participants were informed of the study's objectives and procedures and provided written informed consent. Participation was voluntary, and all responses were anonymized to ensure confidentiality and protect participants' privacy.

This study employed a quasi-experimental design to evaluate the effect of Teacher-Student Collaborative Assessment (TSCA) on academic burnout among students with varying learning styles. This design is especially suitable since students could not be randomly assigned to learning style groups, which were determined using the Kolb Learning Style Inventory (KLSI 3.1). Therefore, the grouping was based on intrinsic learner characteristics rather than artificial treatment conditions. Quasi-experimental designs are widely recognized in educational research as a strong alternative to randomized controlled trials (RCTs) when these are not feasible due to ethical or logistical constraints. In our study, two classes were exposed to the same TSCA intervention over a 3-month period, with all students receiving identical instructional content, taught by the same instructor, using standardized materials. Academic burnout was measured using a validated scale at the beginning and end of the semester, allowing for within-group comparisons of changes over time. To further strengthen the design's internal validity, potential confounding variables—such as instructor bias, curriculum variation, or assessment differences—were controlled. The design allows for causal inference to a reasonable degree while preserving the authenticity of classroom dynamics, which is critical for evaluating pedagogical innovations in real-world educational contexts. Therefore, the quasi-experimental design represents a methodologically sound and contextually appropriate choice for this study.

To minimize potential confounding variables and strengthen the internal validity of the quasi-experimental design, several key controls were implemented across all groups. All three classes (A, B, and C) were taught by the same instructor, thereby eliminating variation due to instructor teaching style, experience, or rapport with students. The instructor strictly adhered to a standardized teaching plan, utilizing the same lecture content, instructional materials, and assignment formats used across classes. Course content was fully synchronized across sections, following a university-approved syllabus for Clinical Molecular Biology Testing. No elective modules or instructor-selected topics were introduced in any of the classes. This ensured that all students, regardless of group, encountered the same curriculum with equivalent academic expectations. The student workload—including class hours, assignments, online tasks, and assessment weightings—was matched across all groups. The TSCA intervention was embedded within this structure but did not increase the overall time or effort required from students. Importantly, the Academic Burnout Scale was administered at identical time intervals across all groups (Week 1 and Week 12), reducing the likelihood that temporal variation or differential exposure could bias outcomes. While random assignment was not feasible due to the nature of educational settings and pre-existing class enrollments, the use of procedural uniformity and environmental consistency helped mitigate the influence of confounding factors. These steps enhance the interpretability of the findings and provide a robust foundation for causal inference regarding the effect of the TSCA model on academic burnout.

### 3.2 Determination of types of student learning styles

The Kolb Learning Style Inventory Scale (3.1-KLSI), designed by David A. Kolb, was used as a tool for measuring different types of learning styles (Kolb and Kolb, 2017). This measurement is based on the learning styles classification model calculated through the KLSI, which locates the respondent's position within a coordinate model. The model identifies four types of learning styles: adaptive, assimilative, decentralized, and convergent. Participants are evenly distributed into these four groups based on their selected style.

The instruments used in this study have demonstrated acceptable measurement quality within the context of Chinese undergraduate education. The Kolb Learning Style Inventory (KLSI 3.1) is based on experiential learning theory and has been widely applied to assess students' cognitive processing preferences. Its four-category model—adaptive, assimilative, divergent, and convergent—has shown stable dimensionality and internal consistency across multiple applications. Similarly, the Academic Burnout Scale for College Students (ABSC) has been designed and adapted for the Chinese higher education context. It captures three key subdimensions: emotional exhaustion, academic inefficacy, and disengagement. In the present study, Cronbach's alpha coefficients for the subscales ranged from 0.82 to 0.89, indicating good reliability and consistency. These instruments are suitable for examining the relationship between learning styles and academic burnout in undergraduate populations. A summary of their psychometric properties and measurement scope is provided in Table 1. Although most participants could be clearly classified into a single dominant learning style, in a few cases where two dimension scores were closely matched, additional confirmation was obtained through further interviews or self-reflection reports. Ultimately, each student was assigned to only one learning style group to ensure mutual exclusivity and analytical clarity.

**Table 1:** Psychometric properties of instruments used in the study.

**Instrument**	**Version**	**Reliability (Cronbach's α)**	**Context**	**Suitability**
Kolb learning style inventory	3.1	0.72–0.81	Undergraduate learning assessments	Commonly applied in cognitive learning style research
Academic burnout scale	Localized college version	0.82–0.89	Emotional and motivational screening for students	Suitable for the Chinese higher education environment

Table 2 presents examples from the Kolb Learning Style Inventory (KLSI 3.1) and the Academic Burnout Questionnaire used in this study. The KLSI includes forced-choice items that classify learning styles across four dimensions. A typical item prompts learners to choose between descriptions, such as relying on intuition or trying things out. The scale demonstrated acceptable internal consistency in this study (Cronbach's alpha = 0.82). Burnout was assessed using a validated 47-item questionnaire that measures emotional exhaustion, achievement, and cynicism. An example item is “I often feel mentally exhausted by my coursework.” This instrument showed high internal consistency (Cronbach's alpha = 0.91). These tools provided reliable assessments of key constructs, supporting the robustness of our findings.

**Table 2:** Examples and psychometric properties of measurement instruments.

**Instrument**	**Sample item**	**Psychometric indicator**
Kolb learning style inventory 3.1 (KLSI)	When I learn, I am best described as someone who… (A) relies on intuition, (B) observes carefully, (C) thinks about ideas, (D) likes to try things out.	Cronbach's α = 0.82
College Students' Academic Burnout Questionnaire	I often feel mentally exhausted by my coursework.	Cronbach's α = 0.91

### 3.3 Detecting the degree of students' academic burnout

The College Students' Academic Burnout Questionnaire Scale served as a testing tool and included two parts: the first part with 20 items and the second part with 27 items. It was based on various dimensions such as emotional state, sense of achievement, behavioral motivation, emotional commitment, ideal commitment, and others. The scale utilized five degrees of compliance ranging from 1 (complete non-compliance) to 5 (complete compliance), indicating that a higher total score reflects a greater degree of academic burnout. Over the 3-month study period, the academic burnout of students with four different learning styles was assessed in the first and third months using the Academic Burnout Scale for College Students (ABSC), comparing academic burnout levels among the different groups before and after the study. Prior to conducting inferential analysis, the data were examined for compliance with the assumptions of parametric testing. The distributions of burnout scores were assessed using the Shapiro-Wilk test, and no severe violations of normality were observed. Homogeneity of variance was evaluated using Levene's test, which supported the use of ANOVA for between-group comparisons. Given that burnout scores were collected at two time points from the same individuals, a within-subjects approach was appropriate for detecting pre-post changes in each group. Therefore, paired-sample *t*-tests were applied to each learning style subgroup. To compare differences in intervention effects across learning style groups, a one-way ANOVA was selected as a robust method for evaluating group-level variation in change scores. These analyses allowed us to assess both individual improvement and the differential responsiveness of learning styles to the TSCA intervention.

Statistical analysis was conducted using SPSS 26.0 to evaluate the impact of the TSCA intervention on academic burnout. Paired-sample *t*-tests were used to examine changes in burnout scores from pre- to post-intervention within each learning style group in Class A and Class B. A one-way ANOVA was conducted to analyze differences in burnout change scores among the four learning styles. Significance was determined at an alpha level of 0.05. Cohen's *d* was reported for within-group effect sizes, while partial η^2^ was calculated for ANOVA. Where applicable, 95% confidence intervals were also reported for the mean difference to quantify the precision of effect estimates. In Class A, the divergent group showed a significant reduction in burnout (*t*(8) = 3.42, *p* = 0.009, *d* = 1.14, 95% CI [2.88, 6.49]), and the adaptive group also demonstrated a reduction (*t*(11) = 2.65, *p* = 0.022, *d* = 0.85, 95% CI [1.24, 5.11]). In Class B, both the divergent (*t*(9) = 3.85, *p* = 0.004, *d* = 1.22, 95% CI [3.27, 6.71]) and adaptive (*t*(10) = 2.47, *p* = 0.033, *d* = 0.78, 95% CI [1.15, 4.96]) groups also showed significant decreases. No significant change was observed for the assimilative or convergent groups in either class, and their confidence intervals included zero. A one-way ANOVA revealed a significant main effect of learning style on burnout reduction (*F*(3, 76) = 4.28, *p* = 0.008, η^2^ = 0.15), confirming differential responsiveness to the TSCA model. Summary results, including test statistics and confidence intervals, are presented in Tables 3, 4. To assess the practical significance of the findings, effect sizes were calculated and interpreted according to established guidelines. For within-group comparisons, Cohen's *d* values ranged from 0.78 to 1.22 in the adaptive and divergent groups, indicating moderate to large effects based on conventional benchmarks (*d* ≈ 0.2 = small, 0.5 = medium, 0.8 = large). These values suggest that the TSCA intervention had a meaningful impact on reducing burnout in these learners, not merely statistically. For between-group comparisons, the partial η^2^ value derived from the one-way ANOVA was 0.15, which falls into the medium effect size range (η^2^≈0.01 = small, 0.06 = medium, 0.14 = large), indicating that a substantial proportion of the variance in burnout reduction can be attributed to learning style differences. In contrast, the assimilative and convergent groups showed small or negligible effect sizes (Cohen's *d* < 0.35), with confidence intervals crossing zero, suggesting limited practical benefit from the TSCA model for these profiles. These effect size interpretations help contextualize the TSCA's effectiveness and provide a nuanced understanding beyond *p*-values alone.

**Table 3:** Paired *t*-test results with 95% confidence intervals for burnout reduction.

**Group**	**Class**	***t*(df)**	***p***	**Cohen's d**	**95% CI (Δ)**	**Significance**
Adaptive	A	2.65 (11)	0.022	0.85	[1.24, 5.11]	*
Adaptive	B	2.47 (10)	0.033	0.78	[1.15, 4.96]	*
Divergent	A	3.42 (8)	0.009	1.14	[2.88, 6.49]	**
Divergent	B	3.85 (9)	0.004	1.22	[3.27, 6.71]	**
Assimilative	A	0.94 (9)	0.368	0.29	[-1.15, 3.21]	ns
Assimilative	B	1.12 (8)	0.293	0.35	[-0.84, 2.87]	ns
Convergent	A	0.87 (9)	0.407	0.27	[-1.05, 2.91]	ns
Convergent	B	0.91 (9)	0.384	0.29	[-0.97, 3.08]	ns

*p < 0.05, **p < 0.01, ns, not significant; CI, 95% confidence interval of mean difference.

**Table 4:** One-Way ANOVA results for burnout change scores across learning styles.

**Source**	**df**	***F*-value**	***p*-value**
Between groups	3	4.28	0.008
Within groups	76	—	—
**Effect size (partial** **η^2^)**	0.15		

Table 5 presents the means, standard deviations, and sample sizes for each learning style group in both Class A and Class B, measured before and after the TSCA intervention. At baseline (pre-intervention), burnout scores ranged from ~ 41 to 46 across groups, with divergent learners generally reporting the highest levels. Post-intervention means declined most notably in the adaptive and divergent groups, consistent with the inferential results. The stability of standard deviations across time points also indicates consistent response behavior. These descriptive statistics provide essential context for understanding the magnitude and direction of change within each group.

**Table 5:** Descriptive statistics of academic burnout by learning style and time point.

**Group**	**Class**	**Time**	***n***	**Mean**	**SD**
Adaptive	A	Pre	24	42.83	6.21
Adaptive	A	Post	24	38.17	5.97
Adaptive	B	Pre	8	43.36	6.48
Adaptive	B	Post	8	39.09	6.12
Divergent	A	Pre	24	45.89	5.44
Divergent	A	Post	24	39.56	4.83
Divergent	B	Pre	8	46.10	6.03
Divergent	B	Post	8	39.20	5.20
Assimilative	A	Pre	24	41.73	5.92
Assimilative	A	Post	24	40.29	5.77
Assimilative	B	Pre	8	42.88	5.86
Assimilative	B	Post	8	41.67	5.61
Convergent	A	Pre	24	43.21	6.34
Convergent	A	Post	24	42.57	6.11
Convergent	B	Pre	8	43.60	6.22
Convergent	B	Post	8	42.69	5.98

The TSCA model was implemented on a weekly basis over the course of a full semester (12 weeks), following a consistent three-stage cycle: pre-class rehearsal, in-class exploration, and post-class extension. In the pre-class stage, students were assigned a preparatory task and a short quiz (typically consisting of two analytical questions) 4 days before each class session. Students were encouraged to complete the assignment within 30 min and submit their answers 1 day prior to class. Group discussions were held online or offline within small, learning style-based teams, requiring active participation and initial peer feedback. During the in-class stage, each session started with a 5-min review of the pre-class tasks, followed by a 15-min collaborative group discussion led by the instructor. This was followed by a teacher-guided teaching phase lasting ~ 70 min, during which student groups were called upon to present their ideas and respond to questions, integrating their prior analysis with classroom input. The post-class extension phase consisted of open-ended reflective questions that required students to connect course concepts to real-life applications. This task was expected to be completed in ~ 30 min and submitted within 2 days. All stages involved student-generated peer evaluations and teacher feedback, which were formally recorded weekly. The active engagement of students was essential at every stage, particularly in co-assessment and reflection tasks, reinforcing the dual role of learners as both evaluators and contributors to the instructional process.

### 3.4 TSCA design in teaching and learning

The overall process of TSCA was carried out through three stages: pre-class rehearsal, classroom exploration, and after-school extension, which were specified as follows:

Pre-class rehearsal: the pre-class task and pre-class test homework (a total of 2 questions) were given 4 days before class. Students discussed the questions in their groups, and each person reported the results. Each student needed to complete the task in 30 min and submit it 1 day before class.

Classroom exploration: in class, the pre-class questions were summarized and explained in the first 5 min. Then, the students, divided into 4 groups, began discussing the questions for 15 min. Each group answered the teacher's questions, and the remaining 70 min were dedicated to normal teaching.

After-school extension: the teacher posed open-ended questions and encouraged students to reflect on real-life connections through online engagement. All four learning style groups communicated with one another, and each student completed the after-school assignment within 30 min, submitting it within 2 days.

**Figure 1:**
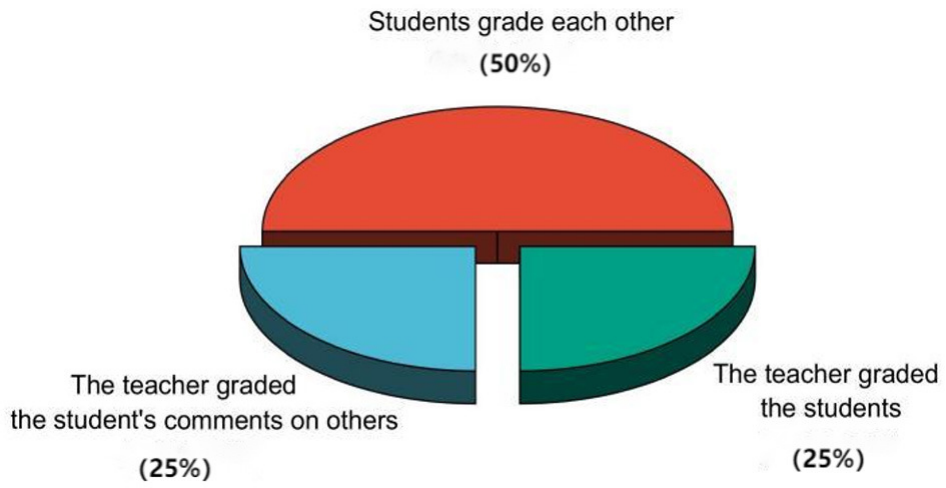
Single-stage scoring component model.

All three stages relied on the same scoring model, as shown in Figure 1, which indicated that a student's grade consisted of three parts: teacher scoring (25%), student peer assessment (50%), and the teacher's evaluation of the student's comments on others' comments (25%). The scores from the three stages were then synthesized into a total grade with the proportions of pre-class preview (25%), in-class exploration (50%), and post-class extension (25%), and this synthesized total was used as the final grade (60% of the overall score). A teacher-student collaborative evaluation form was filled out after the last class of each week to gather scores, along with a student evaluation teacher scale to collect suggestions for improving teaching methods.

For clarity, we provided examples of the tasks and questions designed for each TSCA phase. During the pre-class rehearsal, students were asked to summarize the basic workflow of PCR-based molecular diagnostics and identify two clinical scenarios in which it applies. During the classroom exploration phase, a representative in-class question was: given a case study involving a suspected genetic disorder, how would you interpret the patient's PCR results, and what follow-up tests would you recommend? In the after-school extension, students responded to open-ended prompts such as: reflect on the limitations of molecular diagnostic techniques in personalized medicine. How might TSCA help reduce diagnostic errors in practice? These tasks are designed to promote critical thinking, collaboration, and the application of theoretical knowledge to real-world problems.

## 4 Results

### 4.1 Analysis of learning styles

**Figure 2:**
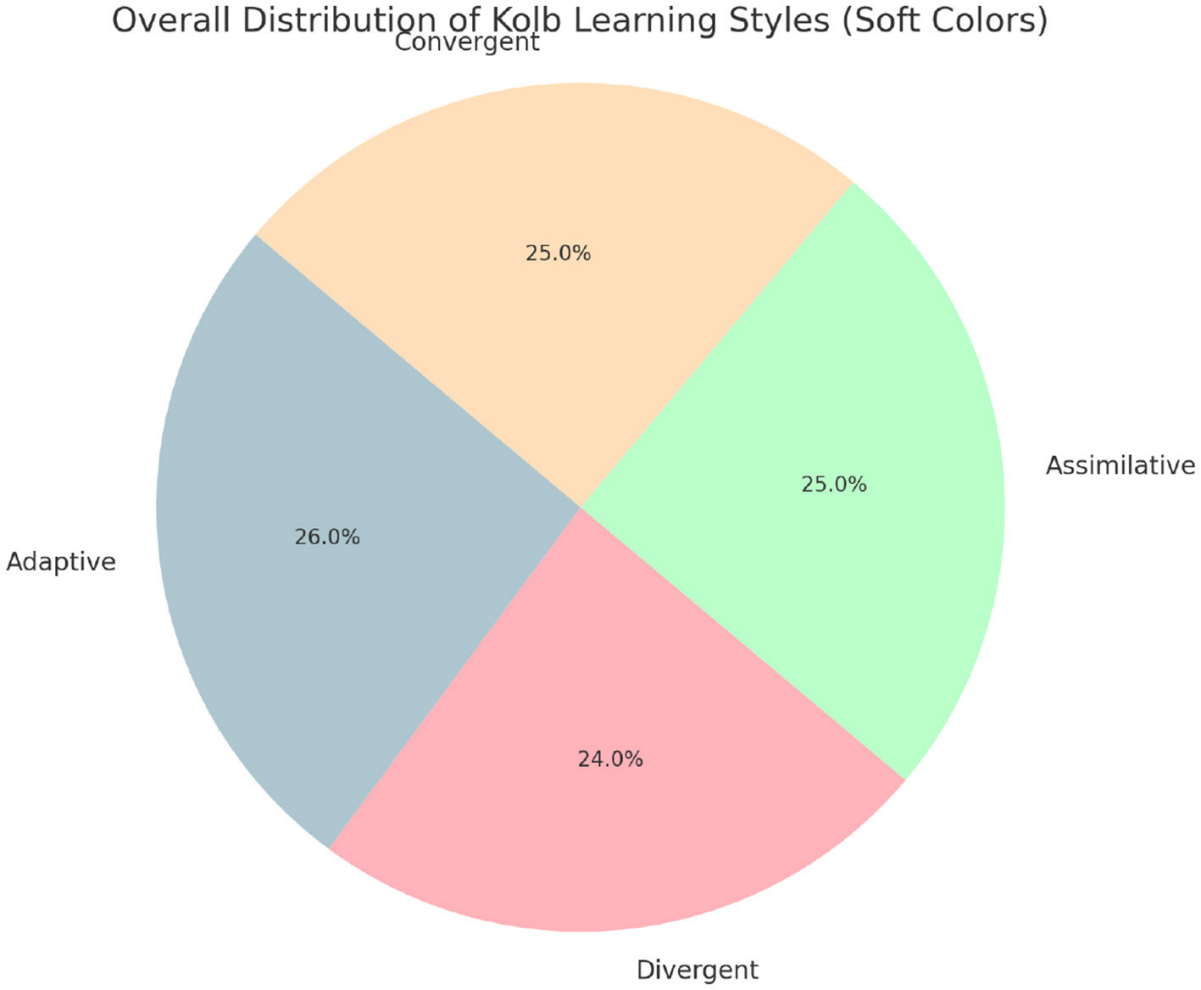
Overall distribution of Kolb learning styles among all participants (aggregated view).

The scores of the two classes in the four stages of the “Kolb learning style scale” differed between male and female, as shown in Tables 6, 7. There was no significant difference between male and female in Class A and Class B (*P*>0.05). According to the Kolb Learning Style questionnaire completed by students in two classes, the distribution pattern of different Kolb learning styles varied in each class. The overall distribution of learning styles among participants was relatively balanced, with each style accounting for approximately one-quarter of the total population. Figure 2 presents this distribution in aggregate form to ensure full confidentiality.

**Table 6:** Kolb scale scores for each dimension in Class A.

**Gender**	**Abstract**	**Active**	**Concrete**	**Reflective**
	**conceptualization**	**experimentation**	**experience**	**observation**
Male	29.76 ± 7.02	29.40 ± 7.09	30.45 ± 7.00	30.62 ± 6.67
Female	29.41 ± 6.30	30.63 ± 6.76	30.91 ± 6.36	29.11 ± 6.28
Total	30.41 ± 6.59	30.33 ± 6.90	30.71 ± 6.62	29.77 ± 6.46

**Table 7:** Kolb scale scores for each dimension in Class B.

**Gender**	**Abstract**	**Active**	**Concrete**	**Reflective**
	**conceptualization**	**experimentation**	**experience**	**observation**
Male	33.57 ± 5.77	30.15 ± 5.02	29.53 ± 6.35	32.31 ± 6.08
Female	30.25 ± 5.26	29.99 ± 5.56	29.36 ± 6.15	31.52 ± 5.22
Total	31.91 ± 5.59	30.07 ± 5.51	29.45 ± 6.22	31.92 ± 5.67

### 4.2 Analysis of changes in academic burnout in different learning style groups

**Figure 3:**
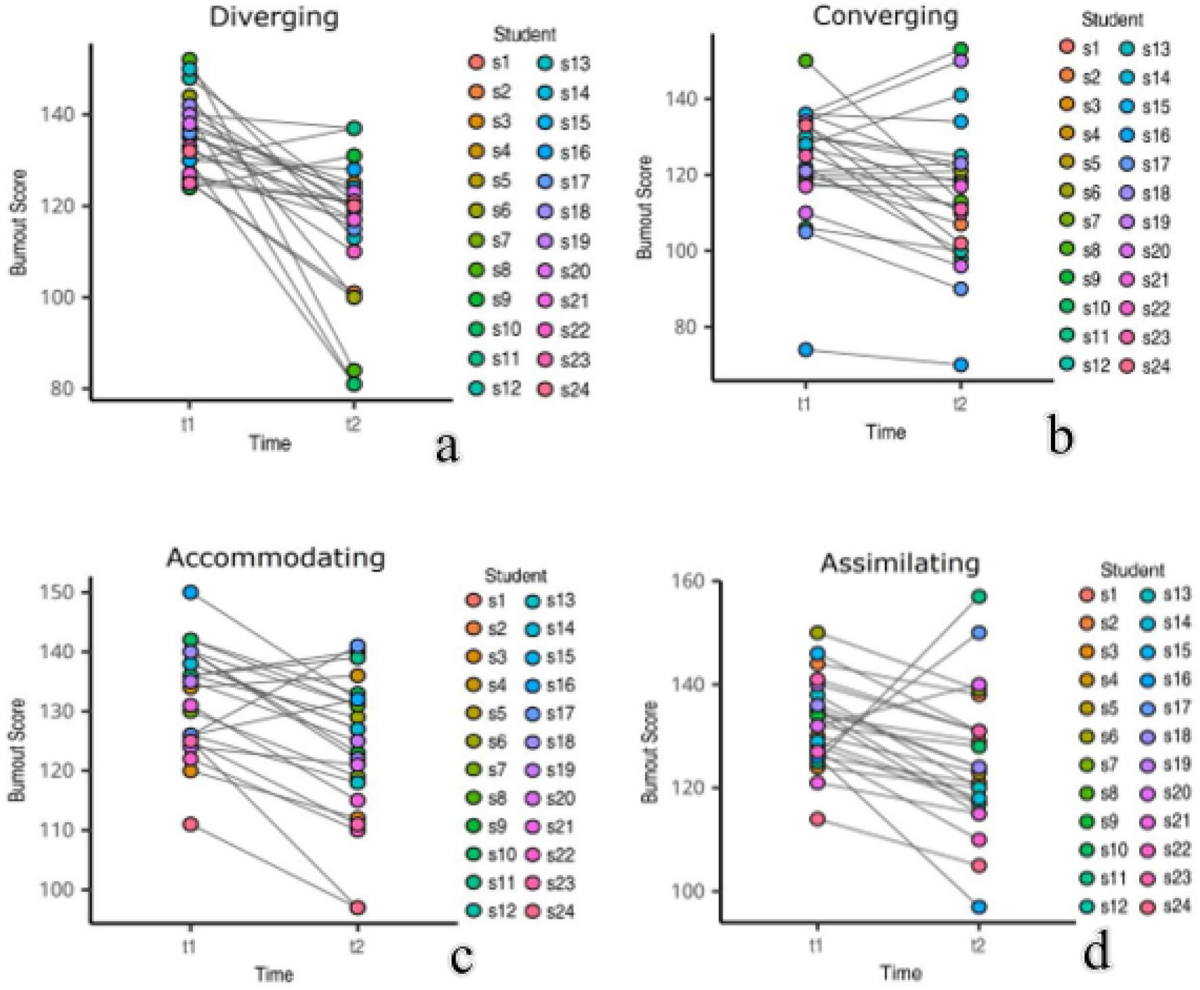
Changes in individual academic burnout across different learning style subgroups in Class A at two points: **(a)** Changes in individual academic burnout in the decentralized learning style group, **(b)** Changes in individual academic burnout in the convergent learning style group, **(c)** Changes in individual academic burnout in the adaptive learning style group, and **(d)** Changes in individual academic burnout in the assimilative learning style group.

**Figure 4:**
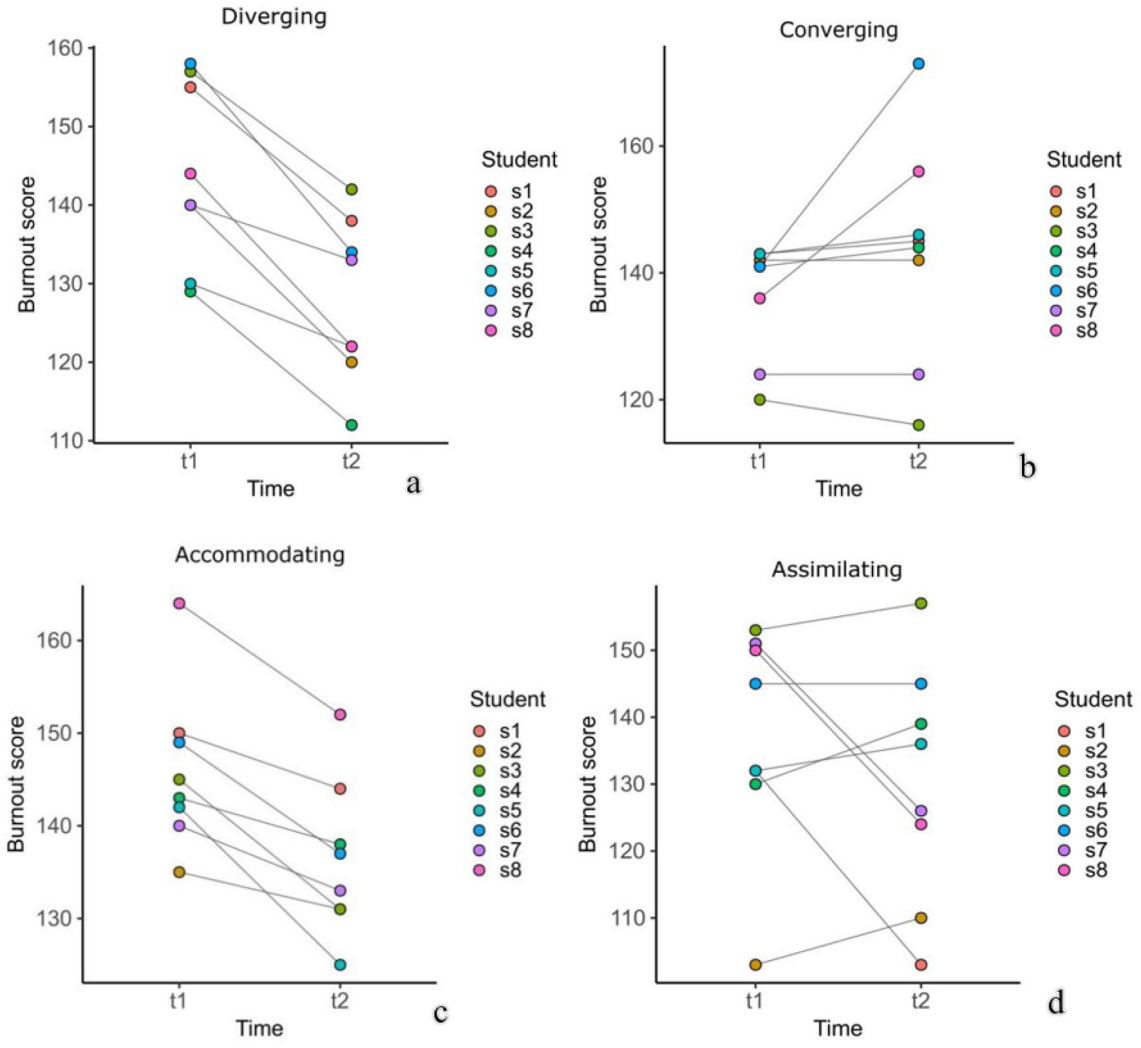
Changes in personal academic burnout across different learning style subgroups in class Bin two points: **(a)** The changes in personal academic burnout within the decentralized learning style group, **(b)** The changes in personal academic burnout within the convergent learning style group, **(c)** The changes in personal academic burnout within the adaptive learning style group, and **(d)** The changes in personal academic burnout within the assimilative learning style group.

The students' academic burnout was evaluated at two points in time: in the early and late stages of the semester's teaching process. The changes among individuals in different learning style groups in Class A are illustrated in Figures 3, 4. Overall, the academic burnout of the four learning style groups decreased after the introduction of the TSCA model. In both classes, the level of burnout among divergent and adaptive learners declined. However, for certain student types, burnout levels increased, with the clustered (aggregated) style group experiencing the greatest rise.

**Figure 5:**
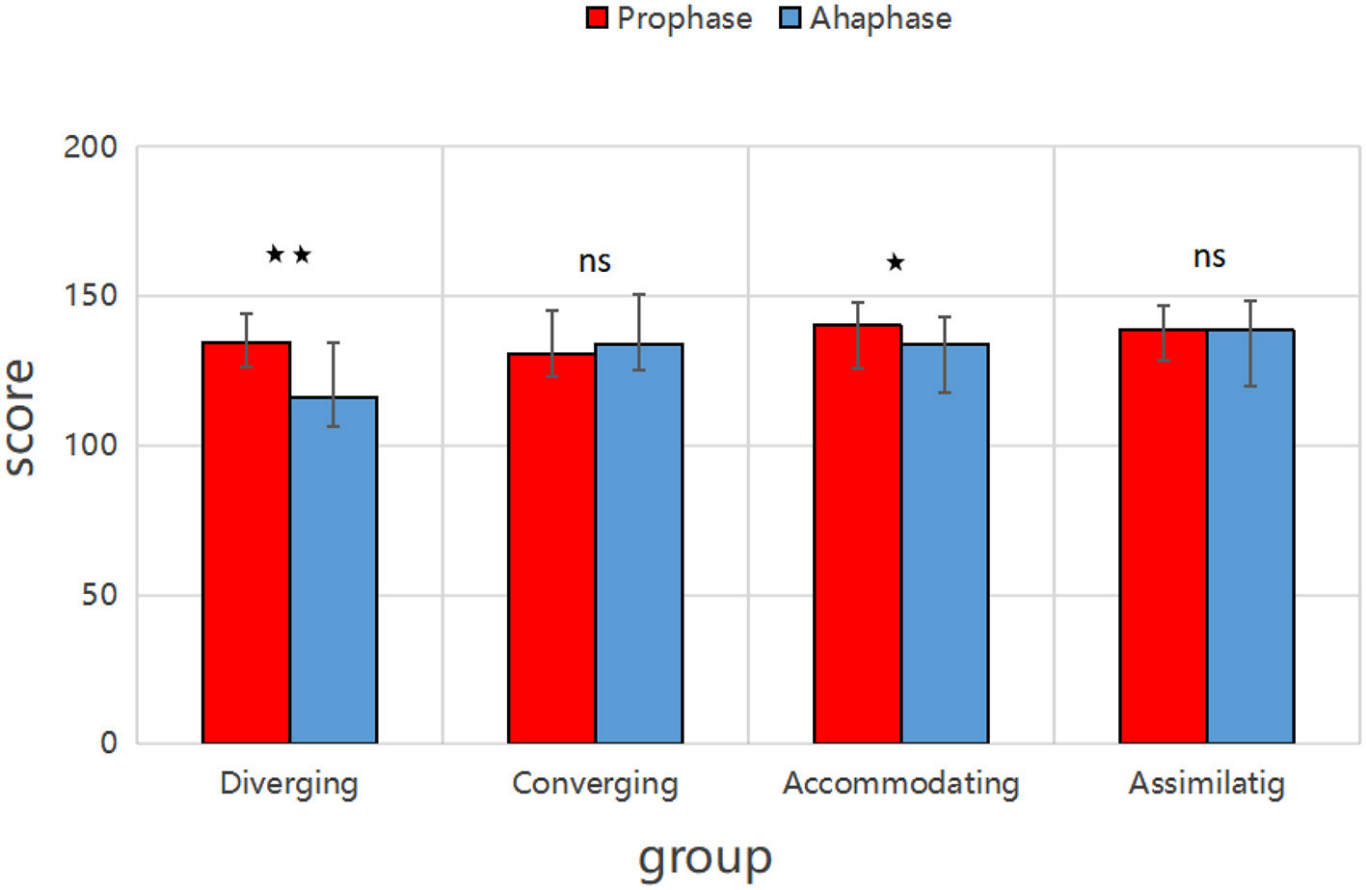
Comparison of academic burnout among different learning styles in two semesters in Class A. In both Class A and B: Diverging group: ⋆⋆ → significant difference in scores between Prophase and Aphase (*p* < 0.01). Accommodating group: ⋆ → moderate significant difference (*p* < 0.05).

In both classes, the burnout levels of students with different learning styles were assessed at two time points. As shown in Figure 5, in Class A, the burnout levels in the divergent group (*P* < 0.01) and the adaptive group (*P* < 0.05) were significantly reduced. In contrast, the aggregated and assimilative groups showed no significant reduction in burnout (*P*>0.05).

**Figure 6:**
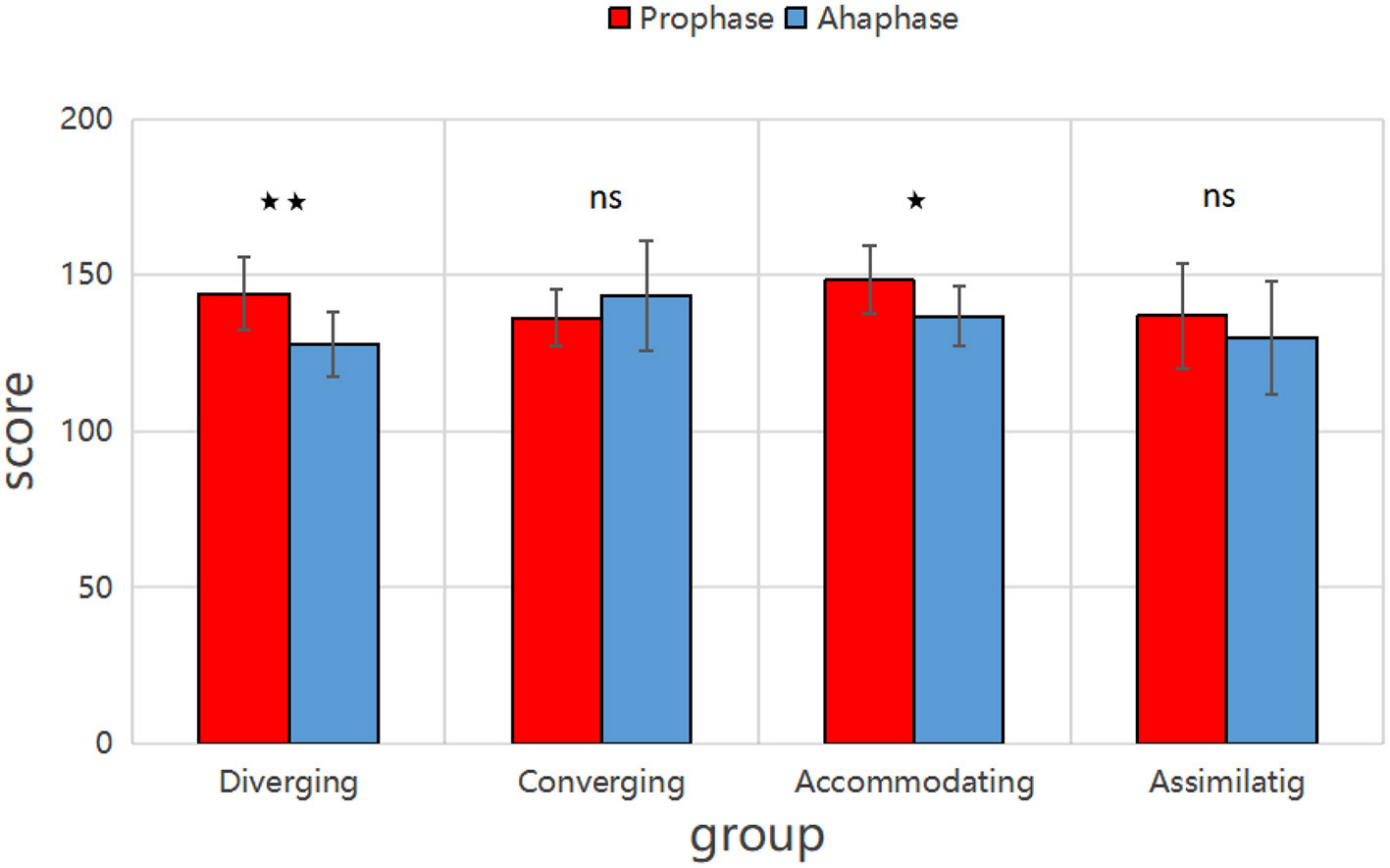
Comparison of academic burnout among different learning styles in two semesters in Class B. Diverging group: ⋆⋆ → significant difference in scores between Prophase and Aphase (*p* < 0.01). Accommodating group: ⋆ → moderate significant difference (*p* < 0.05).

As shown in Figure 6, in Class B, the burnout levels of the divergent group (*P* < 0.01) and the adaptive group (*P* < 0.05) decreased significantly. In contrast, the aggregated and assimilative groups showed no statistically significant change in burnout levels (*P*>0.05).

To strengthen the internal validity of the study and address methodological limitations, a control group (Class C) was introduced. This group consisted of 37 students enrolled in the same course, but they were instructed through traditional lecture-based teaching without exposure to the TSCA model. All three classes—A, B (experimental), and C (control)—were taught by the same instructor using an identical syllabus, schedule, and instructional materials. Academic burnout was measured in all groups using the same validated scale at the beginning and end of the semester. Table 8 presents the distribution of students among the four Kolb learning styles (adaptive, assimilative, divergent, convergent) for each class. The sample sizes are evenly distributed across learning styles, establishing a strong foundation for comparative analysis. By incorporating a control group, the study design allows for direct comparison of pre- and post-intervention outcomes between TSCA and non-TSCA participants, thereby improving causal attribution. Results showed that the reduction in academic burnout among adaptive and divergent learners in the TSCA groups (Class A and B) was statistically significant (*P* < 0.05), while no such reduction was observed in the control group. This finding suggests that the TSCA model has a targeted and style-sensitive impact on burnout alleviation, highlighting its pedagogical value for differentiated instruction.

**Table 8:** Sample sizes and proportions per learning style in experimental and control groups.

**Learning style**	**Class A (TSCA)**		**Class B (TSCA)**		**Class C (Control)**	
	***n***	**%**	***n***	**%**	***n***	**%**
Adaptive	24	25.0%	8	25.0%	9	24.3%
Assimilative	24	25.0%	8	25.0%	8	21.6%
Divergent	24	25.0%	8	25.0%	10	27.0%
Convergent	24	25.0%	8	25.0%	10	27.0%
**Total**	96	100%	32	100%	37	100%

### 4.3 Analysis of grades

**Figure 7:**
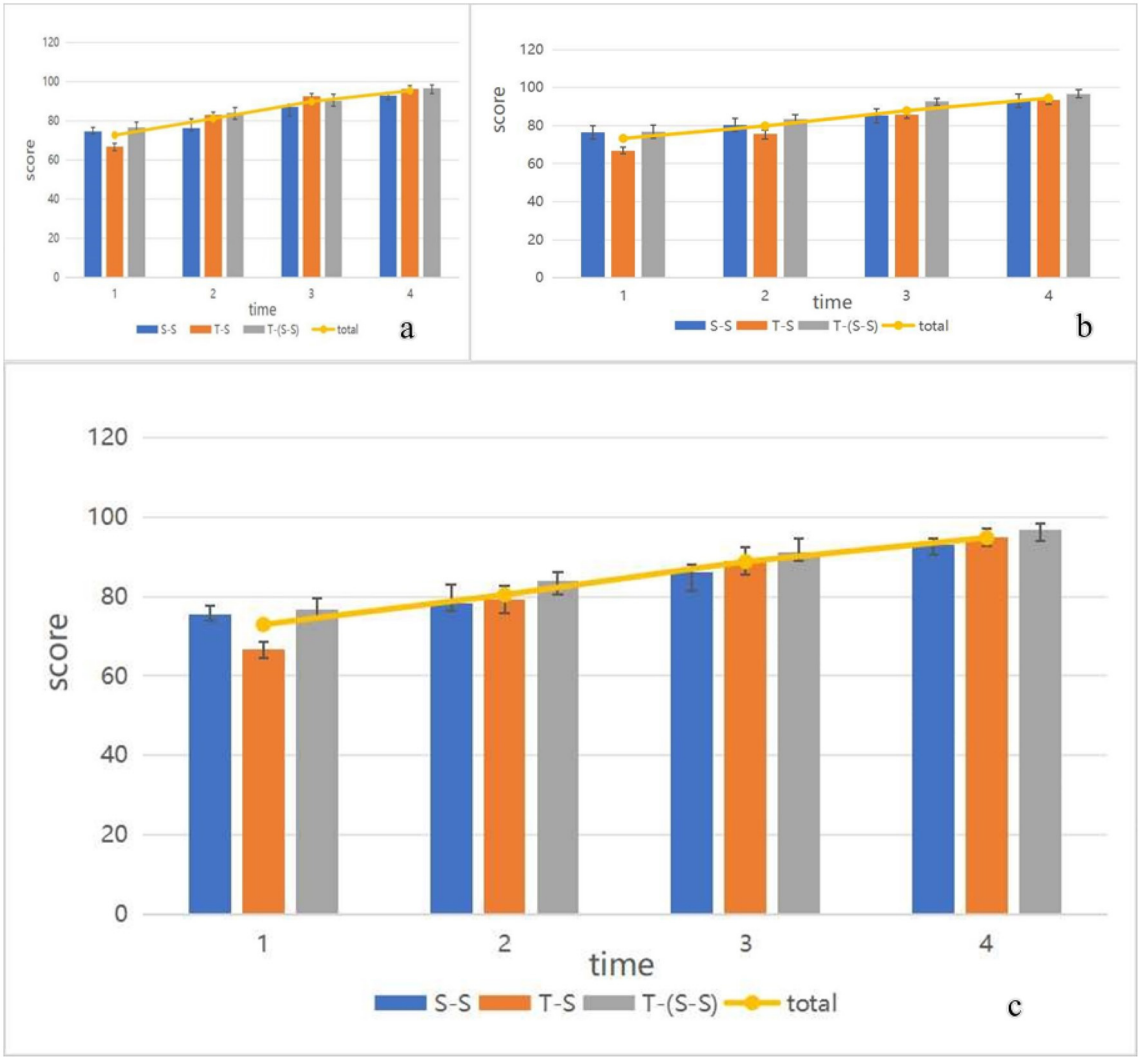
The changes in students' scores at four time points. **(a)** The changes in average scores of students in Class A at four time points. **(b)** The changes in average scores of students in Class B at four time points. **(c)** The changes in average scores of students in Class A and Class B at four time points.

In this study, the students and teachers involved in the TSCA (Teacher-Student Collaborative Assessment) curriculum model were categorized into three components: the teacher's comments on the students, peer evaluations of students, and student self-evaluations. Changes in the average scores for these three components, along with the total average scores for all students across four time points, are depicted in Figure 7, indicating a gradual and consistent improvement from the first to the final evaluation.

**Figure 8:**
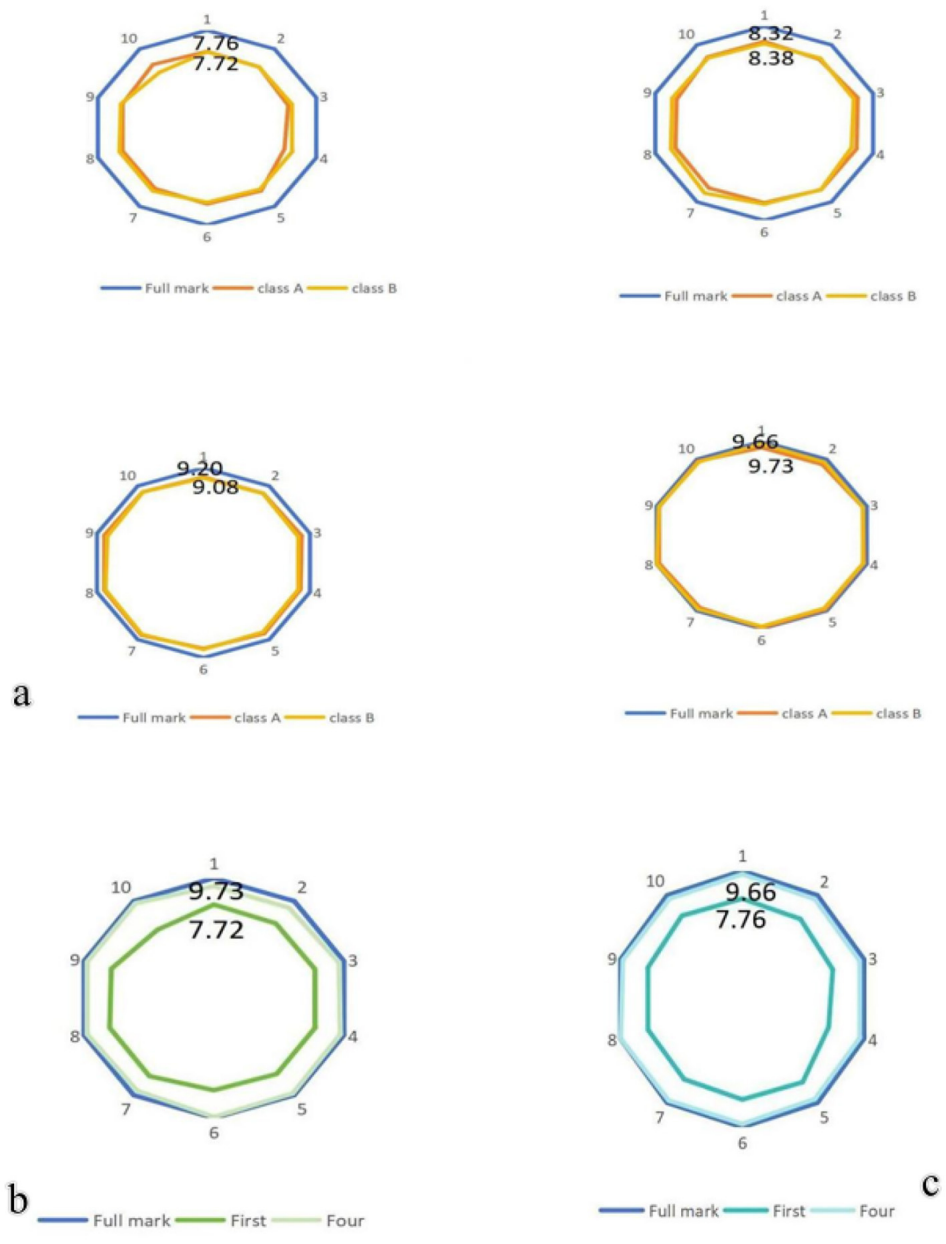
To evaluate the teachers' scores in different classes: **(a)** The teachers' scores in Class A and Class B at four time points. **(b)** The teachers' first and last scores in Class A were compared. **(c)** The teachers' first and last scores in Class B were compared.

Students were asked to complete a 10-item scale, rating each item based on the teacher's performance in teaching. The teacher used the scores from each item to identify strengths and weaknesses in the instructional approach and made targeted improvements or enhancements in subsequent lessons. The assessment scores for both Class A and Class B were recorded at four time points for each item, as shown in Figure 8a. Additionally, the scores from the first and final assessments in Class A and Class B were summarized separately (Figures 8b, c, respectively). These results suggest that student evaluations of teachers may effectively help identify instructional deficiencies in a timely manner and inspire teachers to explore innovative strategies for improving their teaching.

## 5 Discussion

### 5.1 Advantages and outcomes of identifying learning styles for teaching activities

Identifying and clarifying students' learning styles through the characterization of learning behavior based on multidimensional learning data has helped teachers improve their strategies and enhance teaching effectiveness. This approach also promotes the optimization of students' learning strategies and boosts learning efficiency. At the same time, a personalized and specific approach was adopted to establish an academic early warning system and feedback mechanism for students, ensuring their mental health. Specifically, it had the following advantages: personalized teaching, where understanding students' learning styles helped design individualized teaching activities that cater to the needs of diverse learners, thereby enhancing their learning effectiveness. Diversification of teaching methods: utilizing a variety of teaching methods tailored to different learning styles increased the diversity of instruction, enabling all types of students to better understand and absorb knowledge. Increased student engagement: by considering students' learning styles, interest levels were stimulated, enhancing their participation and involvement in teaching activities. Optimizing teaching effectiveness: adjusting teaching strategies and resource allocation based on students' learning styles has improved teaching outcomes, making them more effective and efficient. Enhancement of learning motivation: Teaching activities aligned with students' learning styles fostered their interest and initiative in learning, further stimulating their motivation.

The theory of students' learning styles has gained broad recognition in the education sector. Some teachers have begun integrating learning style identification into their teaching practices by understanding students' personality traits, learning preferences, and knowledge foundations, thereby enabling the implementation of personalized teaching reforms. For example. For example, Song et al. (2024) demonstrated that targeted teaching based on students' learning styles significantly improved enthusiasm, learning outcomes, and performance in rehabilitation nursing practice. Similarly, in a food engineering principles course at a university, instructors designed chapter-specific teaching methods aligned with the Kolb learning style model. This approach demonstrated notable effectiveness, with students' overall scores increasing significantly (Kolb and Kolb, 2006). This teaching mode highlighted a “student-centered” philosophy, where students' awareness of their own learning styles and teachers' understanding of those styles facilitated effective two-way feedback and supported the strategy of “teaching according to students' abilities.” The learning style survey has proven to be a valuable tool in modern college education, enabling educators to identify individual differences and tailor instruction accordingly. In this study, the learning style survey was used as a foundational element in the teaching process to differentiate patterns of academic burnout across various learning styles. At the beginning of the course, academic burnout levels were relatively high across all four learning styles, with burnout slightly more pronounced among students with divergent and adaptive styles.

In interpreting the results, it is critical to relate the findings to established learning theories. The observed improvement in academic burnout among adaptive and divergent learners can be explained through Kolb's experiential learning theory, which emphasizes the importance of concrete experience and active experimentation. These two learning styles thrive in interactive and feedback-rich environments, which TSCA facilitates through structured peer collaboration and self-reflection mechanisms. From a motivational standpoint, TSCA may foster a greater sense of autonomy, competence, and relatedness—key components of self-determination theory—thereby reducing burnout in learners who are more responsive to these factors. However, the study has several limitations. It lacked a control group, making it difficult to isolate the effect of TSCA from other confounding variables. The sample size was relatively small and drawn from specific academic disciplines within a single institution, which restricts the generalizability of the findings. Students were not randomly assigned to TSCA or traditional instruction, which could introduce selection bias. Additionally, the reliance on self-reported measures, such as self- and peer assessments, raises concerns about potential bias and the influence of novelty effects, particularly in the early stages of the intervention. Given these limitations, we advise caution against generalizing the efficacy of TSCA across disciplines or instructional modalities. Future studies should incorporate randomized controlled designs and broader participant pools to validate and refine our findings. Moreover, qualitative insights into students' engagement and cognitive-emotional responses under TSCA could further elucidate its mechanisms of action. The study design included only two measurement points—at the beginning and end of the semester—which limits its classification as a true longitudinal analysis. While paired *t*-tests and one-way ANOVA were suitable for preliminary evaluation, they do not fully capture time-dependent trends or account for individual variability over time. More sophisticated models, such as repeated measures ANOVA or linear mixed-effects modeling, are recommended for future research. These methods would enable a more detailed understanding of the evolution of academic burnout and allow for more accurate estimation of intervention effects over extended periods.

### 5.2 Teacher-student cooperation in evaluating the potential impact of TSCA teaching mode on academic burnout

With the gradual expansion of the scale and number of higher education institutions in China, the connection between compulsory education and higher education has become more frequent. Colleges and universities now provide numerous professional courses with intensive time coverage, making it easier for teachers to observe signs of academic burnout among students. For instance, some researchers have found that nursing majors face tightly scheduled curricula and heavier classroom workloads, leading to a higher prevalence of academic burnout among nursing undergraduates (Kong et al., 2021; Wang et al., 2019). Addressing academic burnout has therefore become an essential focus for educators. In response, some teachers have initiated teaching reforms that emphasize enhancing interaction between themselves and their students, beginning with self-reflection and student engagement.

From the perspective of teacher-student cooperation, the establishment of a cooperative evaluation system among teachers that promotes information sharing, resource sharing, and experience exchange demonstrates the potential to enhance teachers' collective efficacy and teamwork, thereby positively influencing student achievement. TSCA has the potential to boost teachers' collective efficacy and confidence in student development by fostering collaboration and information exchange among teachers, thereby creating a more positive teaching and learning environment. This collaborative assessment model for teaching and learning improves student engagement and the quality of instruction while reducing the incidence of academic burnout. Although TSCA may indirectly affect academic burnout by enhancing teachers' collective efficacy and improving teacher-student relationships, the precise effects require further studies, as well as evaluations of its feasibility and applicability in different educational settings. In this study, the divergent learning style, which typically exhibits higher burnout levels, showed a significant decrease in burnout values after a collaborative teacher-student evaluation of the TSCA instructional model delivery. The adaptive group also showed marked improvement, and the application of TSCA provided the most significant alleviation of academic burnout for the divergent style, while also tending to reduce burnout in the adaptive group. Although there was a numerical decrease in burnout across all four groups, this change was not significant for the convergent and assimilative types. The adaptive group often experienced fatigue due to passive conformity learning in traditional teacher-centered indoctrination, whereas the teacher-student collaborative assessment model provided some initiative and enjoyment. Participation from the diffuse group in the traditional classroom was low, and the TSCA model passively enhanced its participation and met its interactive needs.

It was expected that more TSCA teaching methods would be used in teaching practice to achieve the diversification of evaluation indexes and evaluation methods, which would help diagnose, motivate, and strengthen students' learning processes. Teachers and students formed more efficient learning tools through cooperative mutual assessment. Future studies are expected to explore more impacts of the TSCA model on academic burnout.

The differential effects of the TSCA model across learning styles can be better understood through the lens of Kolb's experiential learning theory and instructional design principles. Divergent learners, for instance, prefer concrete experiences and reflective observation. They thrive in environments that encourage group discussions, open-ended tasks, and interpersonal interaction—features that are central to the TSCA model. By incorporating peer assessment, collaborative problem-solving, and reflective feedback loops, TSCA aligns well with divergent learners' strengths and cognitive preferences. This alignment likely contributed to the significant reduction in burnout observed in this group. Similarly, adaptive learners benefit from hands-on learning experiences and are responsive to dynamic, socially supportive environments. Traditional lecture-based formats often marginalize these learners, but the co-assessment and group-based feedback mechanisms embedded in TSCA may provide the autonomy and interactivity that drive their engagement. This helps explain the observed improvement in academic burnout for adaptive learners as well. In contrast, convergent learners excel in applying abstract concepts through structured problem-solving. They often prefer clarity, direct instruction, and tasks with definite outcomes. The open, student-centered nature of TSCA may introduce ambiguity or reduce perceived efficiency for these learners, weakening its motivational impact. Similarly, assimilative learners prioritize abstract conceptualization and reflective observation but may feel less comfortable with the interpersonal and evaluative aspects of TSCA. The emphasis on mutual peer assessment and discussion-based exploration may not align well with their solitary, theoretical orientation. The lack of significant improvement for convergent and assimilative learners does not necessarily indicate failure; instead, it suggests a mismatch between the pedagogical affordances of TSCA and their dominant learning modalities. Future adaptations of TSCA could include optional structured components or scaffolding strategies to better accommodate these learning profiles.

While the TSCA intervention demonstrated measurable effects on academic burnout, we recognize that external variables may have also played a role in shaping student responses. During the data collection period, students were enrolled in multiple courses, some of which involved intensive workloads or exam preparations. Although these competing academic demands were not formally controlled as covariates in the statistical model, the study's design ensured that all participants—regardless of learning style—experienced similar institutional schedules, thus reducing systematic bias. This study was conducted during a post-pandemic transitional phase, when the university had resumed regular operations but residual stress effects were still observable in some students. To address this, baseline data were collected at the start of the semester, and students' academic burnout was tracked longitudinally instead of cross-sectionally, allowing us to observe changes within individuals over time. No significant policy changes or pandemic-related campus disruptions occurred during the study period. However, we acknowledge that external psychological and academic stressors may exert a background influence on student well-being, and we encourage future studies to incorporate more detailed controls (stress inventories or concurrent academic load tracking) to better isolate the effects of instructional interventions such as TSCA.

## 6 Conclusion

This study aimed to investigate whether the Teacher-Student Collaborative Assessment (TSCA) model could reduce academic burnout among students with varying learning styles in an online medical course environment. Our findings show that TSCA had a statistically significant positive effect on students with adaptive and divergent learning styles, while its impact on convergent and assimilative learners was limited. While these results suggest the potential of TSCA in addressing academic burnout, the study's conclusions should be approached with caution. The sample was drawn from only two classes at a single university within one discipline, and no control group was utilized. These contextual and methodological limitations restrict the generalizability of the findings. Potential biases related to self-assessment and the novelty of the TSCA model were not fully controlled.

Future research should extend the investigation of TSCA to encompass additional academic disciplines and institutions, ideally through randomized controlled trials. Cross-cultural studies could also reveal the degree to which the model is adaptable across various educational settings. Longitudinal designs could help assess whether the observed effects are sustainable over time or influenced by temporary motivational surges. While this study offers preliminary evidence supporting the benefits of TSCA for certain learning styles, more extensive and rigorous investigations are required before recommending its widespread adoption. While the findings support the effectiveness of TSCA in reducing burnout among adaptive and divergent learners, we caution against extending these conclusions to all learner types. The absence of significant effects in the assimilative and convergent groups suggests that TSCA's current design may not align well with their preferred learning modalities. Therefore, the applicability of TSCA should be considered style-dependent, and future refinements should aim to address this diversity through targeted instructional adjustments.

## Data Availability

The original contributions presented in the study are included in the article/supplementary material, further inquiries can be directed to the corresponding authors.
